# Supplementary material for: National TB program shortages as potential factor for poor-quality TB care cascade: Healthcare workers’ perspective from Beira, Mozambique
Source: PLoS One. 2020 Feb 14;15(2):e0228927. doi: 10.1371/journal.pone.0228927 (PMC7021283; doi:10.1371/journal.pone.0228927)
Supplement: S1 File — Supporting information of manuscript “National TB program shortages as potential factor for poor-quality TB care cascade: healthcare workers’ perspective from Beira, Mozambique”. (DOCX) [file pone.0228927.s001.docx]

**Supporting Information**

**Prompts used in the individual in-depth interview and focus group discussions. Supporting information of manuscript “*National TB program shortages as potential factor for poor-quality TB care cascade: healthcare workers’ perspective from Beira, Mozambique*”**

**Approach__________________________________________________________________**

This qualitative study will investigate, from the healthcare workers’ perspective, the factors associated with in-hospital poor-quality TB care cascade and explore local sustainable suggestions to improve in-hospital TB care services. Identifying the factors that impede or facilitate the quality of TB care cascade will contribute to developing ways of reducing diagnostic delay and increasing effective healthcare delivery. As is typical in qualitative research, participant recruitment will continue until data saturation is reached.

The core of the research will involve ethnographic interviews and focus group discussions with TB healthcare staff (auxiliary workers, nurses, laboratory technicians, medical doctors, managers and decision-makers). Ethnographic interview techniques are designed to offer individuals an important space to express their attitudes and concerns as well as their experiences of dealing with disease, treatment, and their personal interaction with institutionalised services. Semi-structured interviews will be developed based on established methodology.

**List of semi-structured questions_______________________________________________**

**Semi-structured interview questions for TB staff**

A selection of the following questions will be asked at any given interview depending on context, position and personal history. These questions are just a guide and not all are asked in any one interview. Each number has a main prompt (in bold) and 2+ following questions.

**Interview questions or prompts**

1. **We would like to know if you have knowledge of the existence of gaps or bottlenecks of TB care cascade within your hospital?**
2. If yes, could you please comment on this (flowchart/steps and components)?
3. Do you think TB care cascade gaps or bottlenecks are serious problem within your hospital?
4. Could you explain why you think so?
5. **What factors or difficulties do you think are associated with the gaps or bottlenecks of TB care cascade within your hospital or in general?**
6. Could you explain why is that so?
7. What would be the three most important factors or difficulties?
8. **How do you think you should locally do (or your hospital directorate or MoH) to overcome the factors or difficulties you’ve mentioned in 2?**
9. What actions/measures do you recommend to colleagues/manager/decision-makers of the hospital (or MoH) in your day-to-day situation on how to improve in-hospital TB care cascade (why you think so)?
10. What is the most important group of colleagues/manager/decision-makers for those recommendations (why you think so)?
11. Do the colleagues/manager/decision-makers follow up the recommendations (why you think so)?
12. **Taking in account into the scarcity of resources (human, material/equipment and financial), what actions/measures mentioned in 3, do you think are acceptable and feasible and that, should be considered/emphasized?**
13. Why is that so?
14. How to proceed to move forward?
15. What is necessary for proper implementation of locally and sustainable high-quality TB care cascade?

**Analysis____________________________________________________________________**

Interview questions will aim to investigate specific themes: TB care cascade gaps or bottlenecks, factors or difficulties, actions or measures to overcome the factors or difficulties, local acceptability and feasibility of the proposed actions or measures. Critical content analysis using inductive analysis and grounded in encoding of transcripts that will be linked to the objectives of the study will be used to analyze the healthcare workers interviews. Quotes that will represent the emerging themes will be selected for inclusion in the manuscript.

**References__________________________________________________________________**

Brouwer M, Coelho E, Mosse CD, Brondi L, Winterton L, van Leth F. Healthcare Workers’ Challenges in the Implementation of Tuberculosis Infection Prevention and Control Measures in Mozambique. PLoS ONE. 2014;9(12).

Kleinman, A., Benson, P. (2006) Anthropology in the clinic: The Problem of cultural competency and how to fix it, *PLoS Medicine*, 3(10), pp. 1673-1676.

Kleinman, A. (1986) *Illness Behaviour: A multidisciplinary model*. Plenum, New York.

Lisboa, M., Fronteira, I., Colove, E. et al. Time delay and associated mortality from negative smear to positive Xpert MTB/RIF test among TB/HIV patients: a retrospective study. BMC Infect Dis. 2019;19(1):18.

Mason, P.H., Roy, A., Spillane, J., Singh, P. (2015) Social, historical and cultural dimensions of tuberculosis, *Journal of Biosocial Science.*

Resnicow. K., Baskin, M., Rahotep, S., Periasamy, S., Rollnick, S. (2003) Motivational Interviewing in health promotion and behavioral medicine settings. *Health Psychology*, 21. 444-451.

Rubak, S., Sandboek, A., Lauritzen, T., Christensen, B. (2005) Motivational Interviewing: a systematic review and meta-analysis. *British Journal of General Practice*, 55, 305-312.

Yin RK. Qualitative Research from Start to Finish. 2nd ed. New York: The Guilford Press, 2016.
